# Therapeutic failure with itraconazole in sporotrichosis due to bariatric surgery^☆^^☆☆^

**DOI:** 10.1016/j.abd.2019.04.015

**Published:** 2020-02-12

**Authors:** Larissa Crestani, Bruno de Castro e Souza, Priscila Kakizaki, Neusa Yuriko Sakai Valente

**Affiliations:** Department of Dermatology, Hospital do Servidor Público Estadual de São Paulo, São Paulo, SP, Brazil

**Keywords:** Bariatric surgery, Itraconazole, Sporotrichosis, Treatment failure

## Abstract

Sporotrichosis is a deep mycosis of subacute or chronic evolution, caused by the dimorphic fungus of the genus *Sporothrix*. The treatment is carried out with antifungal orally or intravenously. Therapeutic success can be affected by several factors, such as altered gastrointestinal physiology by surgery. More and more patients are submitted to bariatric surgeries and the literature for the alterations of the absorption of medications in this context is very scarce. We intend to contribute to a better understanding with this case report of cutaneous-lymphatic sporotrichosis in a patient after bariatric surgery without response to itraconazole treatment, even at high doses.

Sporotrichosis is a deep mycosis caused by the dimorphic fungus of the genus *Sporothrix*. Infection occurs after inoculation and can be confined to the insertion site, reach the lymphatic system or spread.^1^, ^2^, ^3^, ^4^ Treatment is carried out with oral or intravenous antifungals, depending on the clinical form.^2^, ^3^, ^4^

We report a case of lymphocutaneous sporotrichosis in a patient with previous Roux-en-Y Gastric Bypass (RYGB) and unresponsive to itraconazole.

A 39-year-old female patient sought care at another hospital presenting two lesions on the back of her hand and the history of previous contact with a cat knowingly affected by sporotrichosis. Due to her clinical–epidemiological diagnosis, therapy with itraconazole was initiated at 200 mg/day. However, following progression of the disease despite doubling and even tripling the dose of the medication, the patient was led to our hospital.

The examination showed nodules, 2 cm in diameter, painful, discreetly erythematous and fluctuant, following the lymphatic drainage of the right upper limb (Fig. 1). The patient reported that she had undergone RYGB surgery two years before justified by obesity.

**Figure 1:**
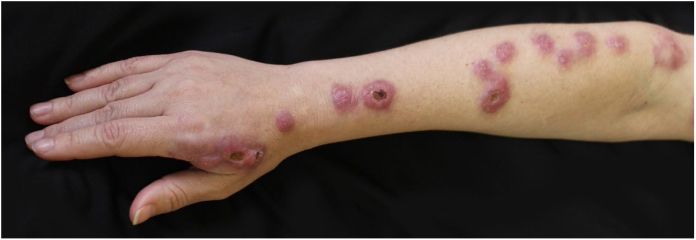
Clinical picture prior to treatment with terbinafine. Nodules approximately 2 cm in diameter, discretely erythematous that followed the lymphatic drainage pathway of the right upper limb. In some lesions there was formation of central ulcers.

In view of the clinical picture and therapeutic failure, we chose to perform a biopsy of a cutaneous lesion, which showed suppurative granulomatous dermatitis and was negative staining for infectious agents. The sporotrichosis diagnosis was confirmed by culture in Sabouraud agar medium and microculture (Fig. 2).

**Figure 2:**
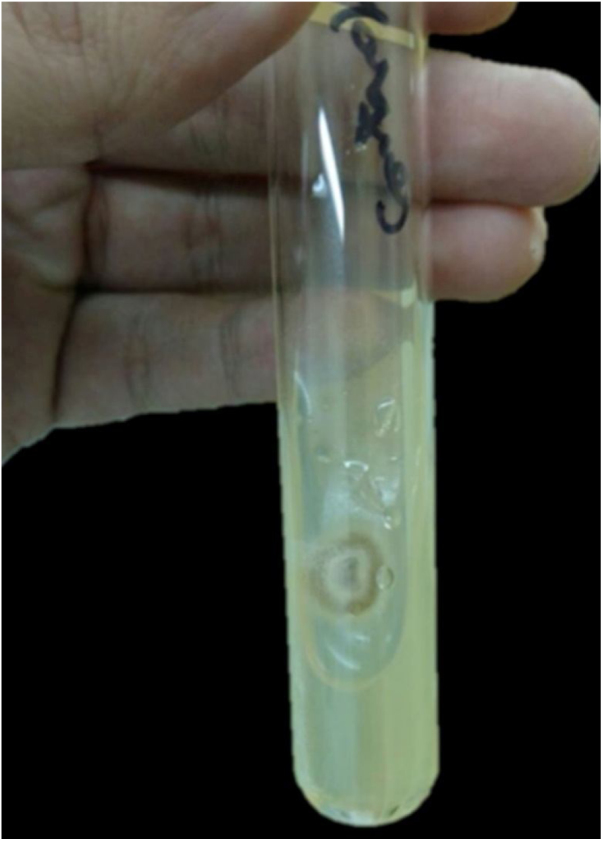
Culture in Sabouraud agar medium.

Due to therapeutic failure, terbinafine 1 g/day was initiated. Following a four-month course, there was clinical cure following complete regression of the lesions (Fig. 3).

**Figure 3:**
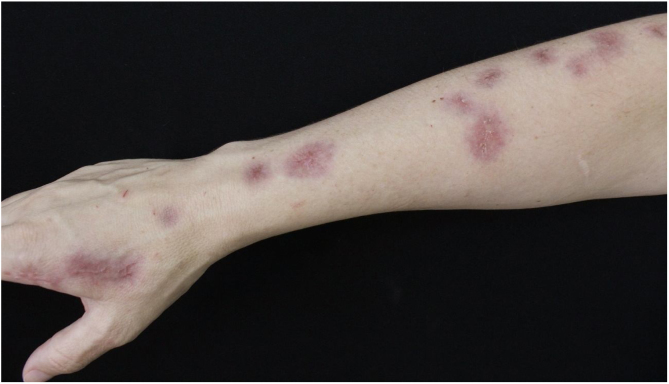
Clinical picture after finishing treatment. Complete regression of lesions.

In the treatment of fixed cutaneous and lymphocutaneous forms, it is recommended itraconazole at 200 mg/day while resistant patients may use up to 400 mg/day of itraconazole, initiate terbinafine 500 mg twice daily or use potassium iodide solution.^2^, ^4^

Itraconazole is a highly lipophilic medicament, requiring acidic media for absorption. Thus, factors that increase gastric pH significantly reduce its absorption.^2^, ^3^ The absorption of terbinafine is not influenced by gastric pH.^2^, ^3^, ^4^

Patients undergoing bariatric surgery have great potential to present malabsorption of medicines, depending on the type of procedure performed, the pharmacokinetics and pharmacodynamics of the medicament. Factors following surgical procedure that influence intestinal absorption are: delayed gastric emptying; decreased exposure of the medicine to intestinal mucosa; and changes in gastric pH.^5^

In RYGB, there is an increase in gastric pH, altering the dissolution and solubility of some drugs, such as itraconazole. In addition to this, lipophilic drugs depend on interaction with bile acids to increase solubility, while gastric bypass decreases the exposition of these medicines with bile acids, thus affecting their absorption.^5^

Therefore, the therapeutic failure with itraconazole is more likely related to the decrease in the absorption of the drug after changes in the gastrointestinal tract's physiology caused by the RYGB, such as the increased gastric pH and the decreased time of contact between the medication and bile acids. Terbinafine's absorption is not influenced by the above factors.

## Financial support

None declared.

## Authors’ contributions

Larissa Crestani: Elaboration and writing of the manuscript; critical review of the literature; critical review of the manuscript.

Bruno de Castro e Souza: Conception and planning of the study; obtaining, analysis, and interpretation of the data; critical review of the literature; critical review of the manuscript.

Priscila Kakizaki: Obtaining, analysis, and interpretation of the data; effective participation in research orientation; intellectual participation in the propaedeutic and/or therapeutic conduct of the studied cases; critical review of the literature; critical review of the manuscript.

Neusa Yuriko Sakai Valente: Effective participation in research orientation; intellectual participation in the propaedeutic and/or therapeutic conduct of the studied cases; critical review of the literature; critical review of the manuscript.

## Conflicts of interest

None declared.
